# Educating students while recruiting underrepresented populations for Alzheimer’s disease research: the Student Ambassador Program

**DOI:** 10.1186/s12909-022-03749-1

**Published:** 2022-10-05

**Authors:** Renée DeCaro, Maureen K. O’Connor, Christina DiTerlizzi, Nana Sekyi-Appiah, John Polk, Andrew E. Budson

**Affiliations:** 1 Center for Translational Cognitive Neuroscience, Veterans Affairs (VA) Boston Healthcare System, 150 South Huntington Avenue, Boston, MA 02130-4817 USA; 2 Edith Nourse Rogers Memorial Veterans Hospital, Bedford, MA USA; 3 Department of Neurology, Outreach, Recruitment, and Engagement (ORE) Core, Alzheimer’s Disease Research Center, Boston University School of Medicine, Boston, MA USA; 4 Diversity and Multicultural Affairs, Boston University School of Medicine, Boston, MA USA

**Keywords:** Alzheimer's disease, Service learning, Participant recruitment

## Abstract

**Background:**

Increasing numbers of patients with Alzheimer’s Disease and related disorders (ADRD) necessitates increasing numbers of clinicians to care for them. Educational programming related to community outreach with older adults may help inspire interest in future ADRD clinical careers, while increasing awareness of ADRD in the community and aiding recruitment of underrepresented participants into research studies.

**Method:**

The Boston University Alzheimer’s Disease Research Center (BU ADRC) created the *BU ADRC Student Ambassador Program,* where medical students, graduate students, and undergraduates interested in medicine completed a curriculum during the academic year that included six educational and three outreach events, including monthly dementia-focused didactic meetings and outreach focusing on Black participant recruitment. A pre-post program survey design was implemented to assess changes in students’ knowledge of and attitudes toward dementia and related disorders.

**Results:**

Between September 2015 and May 2020, thirty-seven students completed the program. Following program completion, students demonstrated increased knowledge of dementia and willingness to work with patients with dementia, as well as more positive attitudes toward patients and the role of empathy in physician practice. In terms of recruitment benefits, the students helped the BU ADRC reach older adults from underrepresented groups who could serve as participants in future research studies.

**Conclusions:**

The BU ADRC Student Ambassador Program can serve as a model for other clinical research programs who wish to encourage students to consider a career in a specific field. In addition, this model has the potential to increase enrollment of participants to research studies. We discuss limitations of our initial efforts and directions for future work to quantify the anticipated benefits for student education and participant recruitment.

**Supplementary Information:**

The online version contains supplementary material available at 10.1186/s12909-022-03749-1.

## Introduction

Approximately 5.8 million people in the United States are living with Alzheimer’s disease (AD), a number projected to double by 2050 [1]. There is a shortage of geriatric, neurologic, and psychiatric healthcare professionals to care for this population [1]. Perceived barriers in the diagnosis and management of dementia contribute to the lack of interest in pursuing geriatric medicine [2]. Medical education is a crucial means to target gaps in dementia knowledge, increase relevent competencies, change students’ perspectives, foster positive attitudes, and garner interest in these careers [2].

Medical education aimed to increase students’ interest in pursuing geriatrics, neurology, and psychiatry can take several forms; foremost among them are formal curricula (e.g., classes and workshops) and service learning programs (e.g., senior mentor programs). Various classes and workshops have led to students’ increased knowledge of and more positive attitudes toward aging; though it is less clear if such programs influence intent of pursuing careers in clinical geriatrics [3–8]. Beyond these, senior mentor programs also have been shown to increase students’ knowledge, attitudes, and interest [9]. Common among these approaches is the primary goal of changing students’ perspectives and competencies.

Unique to service learning programs is the means of advancing not only student education, but also the mutual benefit to the recipients or partners in the program [10]. For example, in senior mentor programs, a student and older adult engage in a learner-mentor relationship where the student learns from the older adults’ experience and perspective, and the older adult can benefit from a new social relationship [9]. We developed an educational program to achieve student learning goals plus an additional aim: recruitment of underrepresented populations in Alzheimer’s disease and related disorders (ADRD) research.

Black participants remain underrepresented in AD research despite their increased incidence of AD relative to non-Hispanic Whites [11]. Increased representation is critical in order to understand differences between Blacks and non-Hispanic Whites in the risk factors for and clinical manifestations of AD as well as delays in the diagnosis and treatment of AD in Black individuals [11]. There are several, multifaceted barriers to and considerations in Black participant recruitment–including the societal racism and historical mistreatment of Black individuals in research studies [12]. This complex issue necessarily warrants a multipronged approach. An initial, potentially beneficial step that may improve research-Black community relationships is increased community presence and engagement [12]. Thus, a program combining student engagement and participant outreach in Black communities may improve student interest in geriatric healthcare *and* older adult interest in AD research in the communities that need it most.

In 2015 the Boston University Alzheimer’s Disease Research Center (BU ADRC) combined medical education and participant recruitment by creating the Student Ambassador Program. We modified the BU ADRC Partnering in Alzheimer’s Instruction Research Study (PAIRS) Program [13], a service-learning program in which students received three hours of lectures and met monthly with a volunteer with early-stage AD. Post-program, students demonstrated increased knowledge of dementia. In the Ambassador Program, the educational topics remained the same, but instead of engagement with one AD volunteer, students participated in outreach activities where they interacted with many older adults in the community. We hypothesized that students’ participation in educational and recruitment activities would increase their knowledge of ADRD and increase positive attitudes toward dementia broadly and older adult populations, in addition to directly assisting in outreach and recruitment efforts.

## Method

### Program design

#### Student Ambassador recruitment

The Program was advertised on departmental websites and social media, in emails to BU medical students, and at in-person job fairs. Interested students completed a detailed application. In this application, students answered questions about themselves (contact information, school year and affiliation), uploaded a resume, and wrote short essays about 1) their previous interactions (if any) with someone with ADRD, 2) their anticipated commitments, outside of coursework, during the academic year, and 3) their interest in the Ambassador program (300 words maximum). Students with complete applications attended an orientation that included an overview of the program and its mission. Interested students signed up for the program by completing a contract agreeing to fulfill program expectations. A blank contract is included in Supplement 1.

#### Program expectations

During the academic year, students were required to attend at least four monthly program didactics, three outreach events, one Community Action Council meeting, and one BU ADRC lecture. Students completed four surveys at the beginning and end of the program, and a reflection paper.

#### Program details

The program took place over the course of eight months during the academic year (e.g., between October and May), where students met monthly for program didactics. These one hour meetings included topics such as the science of the aging brain, information related to AD and other dementias (e.g., pathologies, risk factors, protective factors), research and clinical trial overviews and the importance of recruitment of diverse communities to these. Each one-hour didactic was led by authors AEB or MKO and could feature guest lecturers covering other topics related to AD (e.g., caregiver burden).

Students were also trained to administer brief cognitive screening measures in order to participate in memory screenings (overseen by a neurologist or neuropsychologist) at community outreach events. During the meetings, students discussed and processed their experiences at prior outreach and recruitment events. Methods to improve communication with aging adults in these communities were reviewed.

Recruitment events were hosted at senior and community centers, churches, YMCAs, and libraries in and around Boston. At events, students spoke with community members about their memory concerns, provided information about the ongoing research at the BU ADRC, and encouraged participation in research. Students could attend any 3 events in order to meet their minimum program commitment. Community attendees at the events expressed interest in participating in research by signing up on a contact sheet.

Community Action Council meetings with community leaders included presentations, discussions, and specific initiatives related to the recruitment of underrepresented populations, primarily the Black population in Boston. Held monthly at either the VA Boston Healthcare System or Boston University, the BU ADRC lecture series featured talks from local, national, and international experts in ADRD and related fields.

Pre- and post-program measures were administered via SurveyMonkey (San Mateo, California). Students also completed a two-page reflection paper at the end of the program, where students considered their knowledge of ADRD before and after the program, their experience recruiting and volunteering in the community, and how their participation in the program would affect their training and future work. For fulfilling their commitments, students received a letter signed by BU ADRC Directors as proof of participation in the program for possible inclusion in composite letters from Deans for applications to medical school or residency programs. All students provided assent to allow their de-identified program data to be used for research purposes, in accordance with the Boston University Institutional Review Board determination that this educational research was exempt from written consent.

### Students

Between September 2015 and May 2020, five cohorts completed the one-year program. Fifty-seven students signed the contract outlining program expectations; thirty-seven students (65%) completed the program requirements. Approximately 31% of the total applicant pool were medical students, 6% were psychology graduate students, and the remainder (63%) were undergraduates interested in medicine and/or pursuing a pre-med track. We speculate that reasons for attrition included competing schoolwork demands and scheduling conflicts. In addition, eleven students (55% of total attrition) were from the program year 2019–2020, which likely related to the COVID-19 pandemic that halted in-person events.

Due to the retrospective nature of this report, no demographic information was collected of individuals who participated in the program. However, in order to sample student characteristics, we distributed an anonymous survey to former and current program participants on January 8, 2021. Students answered closed-set questions regarding their program year, academic background while in the program, race and ethnicity, and whether either (or both) of the following applied to them: individual with a disability, individual from a disadvantaged background. Note that definitions for latter categories were from the National Institutes of Health and these definitions appeared on the survey. Students were also asked to freely report the following information: academic background, current field/discipline, age and gender.

In all, 20 students (54%) responded to our request. Of these, the majority were female (female, 90%; male, 10%) and were undergraduates when they participated in the program (undergraduate, 50%; graduate, 25%; medical, 20%; other, 5%). Free report responses to academic background reflect the variety of student participation. Undergraduates were Neuroscience, Medical Sciences, Biology, and Epidemiology majors. Graduate students reported concentrations in Psychology, Occupational Therapy, Medical Sciences, Public Health, and Neuroscience. Medical students were either in their first year (*N* = 3) or fourth year (*N* = 1). In terms of race and ethnicity (*N* = 19), individuals could select multiple options and the selections were as follows: Asian, 10; Black or African American, 1; White, 10; Hispanic or Latino, 3. Three individuals indicated that they were from a disadvantaged background.

### Measures

We administered three surveys: the Jefferson Scale of Physician Empathy (JSPE) [14], the Dementia Attitudes Scale [15], and the Medical Condition Regard Scale (MCRS) [16]. The JSPE measures students’ perceptions of the importance of physician empathy towards patients. The Dementia Attitudes Scale measures students’ comfort level and attitudes toward older adults with ADRD specifically, whereas the MCRS measures students’ willingness to work with patients with dementia generally (e.g., “*Working with patients with dementia is satisfying”*). Both the JSPE and Dementia Attitudes consisted of 20 statements measured on a 7-point Likert scale. The MCRS consisted of 11 statements measured on a 6-point Likert scale. Higher scores corresponded to greater belief in the importance of physician empathy, more positive attitudes toward older adults with dementia, and greater willingness to work with patients with dementia, respectively. We measured another outcome measure of knowledge of dementia using a 64-item quiz implemented previously [13], included in this paper in Supplement 2.

Students completed a two-page reflection paper at the end of the program, where they wrote about their knowledge of AD before and after the program, their experience recruiting and volunteering in the community, and how their participation in the program would affect their training and future work. The reflection assignment was a learning activity and was not scored. As not all papers were available, select quotes are used only to illustrate points in the general discussion and should not be considered outcomes of the present research.

## Results

### Pre- and post-program surveys

We tested changes in sum total scores on the surveys in a series of paired-samples t-tests. Students demonstrated significant gains from pre- to post-program on the JSPE, *t*(36) = 3.40, *p* < 0.01, *d* = 0.56, the MCRS, *t*(36) = 3.21, *p* < 0.01, *d* = 0.53, and the Dementia Attitudes Scale, *t*(36) = 8.14, *p* < 0.001, *d* = 0.75. Each was characterized by a medium effect size (Table 1). Of our measures, only regard for medical conditions and dementia attitudes were significantly associated at pre-test, *r* = 0.47, *p* = 0.003. The greater an individuals’ regard for AD and related disorders (e.g., willingness to work with AD patients), the more positive attitudes they held about the disease and its course.

**Table 1:** Pre-post program measures collapsed across program years 2015–2020 (*N* = 37)

	Possible range		Pre-Test Mean (*SD*)	Post-Test Mean (*SD*)	*M*_Diff_	95% CI *M*_Diff_	
	Min	Max					
JSPE	20	140	118.46 (*12.60*)	122.0 (*12.32*)	3.51	[1.42,	5.61]
MCRS	11	66	54.03 (*6.23*)	56.62 (*5.45*)	2.60	[0.95,	4.24]
Dementia Attitudes	20	140	112.22 (*10.95*)	126.62 (*15.25*)	14.41	[8.14,	20.7]
Dementia Knowledge^a^	0%	100%	68.13% (*9.07*)	75.09% (*8.37*)	6.96	[4.56,	9.37]

*Abbreviations*: *JSPE* Jefferson Scale of Physician Empathy, *MCRS* Medical Condition Regard Scale

^a^*N* = 35

### Changes in knowledge of dementia

As with the survey measures, pre- and post- scores on the knowledge test were subjected to a paired-samples t-test. Two students had incomplete data and were excluded from these analyses. Students made significant gains in total test accuracy, *t*(34) = 5.88, *p* < 0.001, *d* = 0.99 (Table 1). As with previous research [13], students improved in some topic knowledge at greater rates than others. For example, students improved in their knowledge of the diagnosis of AD by 35.1% and the disease’s prevalence in persons over 65 by 24.3%. They also demonstrated increased knowledge of the risk factors for AD including body fat in middle adulthood and low education (increased accuracy by 27.0% and 21.6%, respectively).

### Outreach events

We compared data on outreach events from the year preceding the Ambassador program to the years following the initiation of the program. Data on events held in diverse communities was available for three years preceding the beginning of the Ambassador Program (6 in year 2012–2013; 4 in year 2013–2014; 7 in year 2014–2015), averaging 5.67 events (*SD* = 1.25). Since 2015, we hosted an average of 13.6 events (*SD* = 4.76) in communities reaching a diverse population and an average of 59 Black individuals indicated interest in becoming research participants each year (*M* = 59.4, *SD* = 8.59) (Table 2). Ambassador attendance data are not available for each event; however, we note that at least one Ambassador was present at each event to assist with recruitment.

**Table 2:** Recruitment data for program years, July 2015 to June 2020

Year^a^	Total Ambassadors Each Year	Total Events	Events in Diverse Communities (% of Total)	Black Participants Interested
	*N*	*N*	*N* (%)	*N*
2015 – 2016	7	28	6 (*21%*)	57
2016 – 2017	9	23	17 (*74%*)	60
2017 – 2018	10	54	13 (*24%*)	72
2018 – 2019	6	63	20 (*32%*)	60
2019 – 2020	5	25^b^	12 (*48%*)	48

^a^The Ambassador year corresponds to the academic year. Recruitment data were gathered from July 1^st^ of the calendar year to June 30^th^ of the following calendar year

^b^Total number does not include those events that were cancelled due to COVID-19

## Discussion

The Ambassador Program was initiated in 2015 to combine two previously separate aims: increasing student knowledge of ADRD through service-learning *and* increasing recruitment of underrepresented groups in AD research. Following program participation, students demonstrated increased knowledge of dementia. These knowledge gains, while modest, were consistent with other service-learning programs [13]. Moreover, Ambassador Program requirements were themselves modest—consisting only of six educational meetings (4 didactics, 1 council meeting, 1 lecture) and three recruitment events which could be flexibly attended. Modest aims and flexible attendance are important in the implementation of service learning, as students’ time is limited [10]. Further, students’ selection of which lectures and educational events to attend carries advantages from a self-regulated learning perspective, where student autonomy is linked to better learning outcomes [17]. A possible next step could entail tracking attendance for specific events to determine whether any were more strongly associated with improvements in student outcomes. Indeed, comprehensive tracking of all aspects of the program (e.g., recruitment of students, student demographics, recruitment of community members to actual studies, and longer follow-up of student participants) may provide additional insights into program efficacy.

In addition to knowledge gains, students demonstrated increased positive attitudes and regard for patients with a diagnosis of dementia. Importantly, the scales employed in the present study (Dementia Attitudes and the MCRS) include items assessing perceptions of the treatment and treatability of dementia broadly [15, 16]. Increasing positive perceptions of AD diagnosis specifically and dementia management directly addresses these specific perceived barriers to involvement in geriatric care [2]. Exposure is seen as an essential means of increasing these positive attitudes as well as empathy, as seen in the following student reflection paper quote:

*The people with whom I interacted were no older than my parents. In the future, I will strive to put myself in the shoes of my patients by employing empathy.*

Here, the student reflects on the importance of empathy. We consider this finding particularly important because it has not been widely investigated in the context of service learning programs, but carries far-reaching implications—as empathy is beneficial for every medical specialty. Another also illustrates this point:

*Cumulatively, my time with the Boston University AD Center Ambassador Program has been a fruitful one, teaching me lessons both in the classroom and through the community. Looking ahead to my second year of medical school and beyond, I will remember these moments and believe they have changed the way I approach the elderly, aging, and research. While a challenging subject, I think regardless of whatever specialty I choose, I will need to continue to further my understanding of aging and dementia so that I can better serve the needs of my patients and their families.*

Beyond student outcomes, we speculate that the Ambassador Program increased our ability to reach and recruit participants in the underserved and underrepresented Black communities of Boston, likely because the students’ help enabled us to plan and execute greater numbers of events, and because the students increased the number of ADRC representatives available at these events to engage one-on-one with potential participants. To directly address recruitment barriers, the purpose, importance, and value of AD research to Black communities needs to be effectively and empathetically conveyed [12]. Increased community presence and participation, made possible by the students, represents an important step in addressing such communication gaps:

*Overall, I came away with a sense that there is both a lot going on in the way of creating resources for patients with dementia and their families, and a gap in education and understanding in the wider community.*

Through this service learning, students themselves gain a greater appreciation for the needs of the community:

*Attending recruitment events, including the Essentials of Living and Aging Collaborative in Dorchester, enabled me to interact with the patient populations. Working in the community reminded me that no socioeconomic background, race, or gender could escape AD.*

### Limitations

Several considerations are important when extrapolating findings from the current study. First, as was described in the program details section above, program offerings (e.g., meetings and presentations) varied slightly from year-to-year, due to student interests, available presenters, and the evolving research. In the future, tracking attendance for specific events could determine whether any were more strongly associated with improvements in student outcomes. Second, the core element of the program was community outreach and the administration of the cognitive assessments, and these aspects were foundational to students’ increasing competencies and changes in attitudes. Our student numbers did not allow us to analyze whether subgroups of students (e.g., pre-clinical vs. clinical phase) may benefit differently from this experience. Third, although we tracked student outcomes and participant recruitment, future studies could potentially study the educational benefit provided to the underserved and underrepresented communities targeted by the outreach events.

## Conclusions

In summary, by tying the educational aims of our service learning program to the aims of the BU ADRC’s mission to increase research partication of underrepresented groups, we created the BU ADRC Student Ambassador Program. We hope that programs like this one will increase research participation of underrepresented groups in AD and other diseases. We believe such initiatives will ultimately lead to reductions in healthcare disparities. We further hope that programs like these will inspire medical students to pursue professions in neurology, psychiatry, and geriatrics to increase the number of clinicians with ADRD expertise. Lastly, we believe that these typs of programs will increase overall physician empathy, thereby improving quality of life for all older adults, regardless of race, ethnicity, and background.

## Supplementary Information

**Additional file 1. **Ambassador Program Student Contract.

**Additional file 2. **Boston University Alzheimer's Disease Center PAIRS Program Dementia Knowledge Test.

## Data Availability

The data supporting student learning outcomes and conclusions is available from the corresponding author upon request.

## Abbreviations

AD: Alzheimer’s Disease
ADRD: Alzheimer's Disease and Related Disorders
BU: Boston University
BU ADRC: Boston University Alzheimer’s Disease Research Center
CI: Confidence Interval
IRB: Institutional Review Board
JSPE: Jefferson Scale of Physician Empathy
MCRS: Medical Condition Regard Scale
NIA: National Institute on Aging
NIH: National Institutes of Health
ORE: Outreach, Recruitment, & Engagement
PAIRS: Partnering in Alzheimer’s Instruction Research Study
SD: Standard Deviation
VA: Veterans Affairs
YMCA: Young Men’s Christian Association
