# Supplementary material for: Educating students while recruiting underrepresented populations for Alzheimer’s disease research: the Student Ambassador Program
Source: BMC Med Educ. 2022 Oct 5;22:707. doi: 10.1186/s12909-022-03749-1 (PMC9533970; doi:10.1186/s12909-022-03749-1)
Supplement: Supplementary file 1 — Additional file 1. Ambassador Program Student Contract. [file 12909_2022_3749_MOESM1_ESM.pdf]

Ambassador Program Student Contract

Program Goals:

- To educate BU students about AD and related cognitive impairment,
- To familiarize students with the Boston community and provide the tools and opportunity for students make a difference in research recruitment,
- To improve student communication skills when interacting with individuals with cognitive impairment and potential research participants,
- To introduce students to research and career opportunities in geriatrics, neurology, and related fields, and
- To provide individuals with AD and related cognitive impairment with the opportunity for informal interaction with a student.

Commitments:

The program will run from October through May. At the start of the program, there will be an hour orientation to AD, which will include information on communicating with individuals affected by AD. At the conclusion of the program, students will complete a short reflection paper summarizing their Ambassador program experiences. All student participants will also have a one-hour monthly meeting with other student participants and Ambassador program faculty and staff to share their experiences and discuss any questions that might arise. Periodically, guest speakers will be invited to the monthly meetings to lecture on AD related topics.

As a student participant, I will:

- Complete the pre and post surveys that were sent to you via survey monkey.
- During the academic year I must attend at least *three* outreach and recruitment events/activities.
- I will attend monthly didactic meetings. I will attend at least *four* out of the eight monthly meetings.
- I will attend at least one Community Action Council meeting.
- I will attend at least one BU ADC lecture that is held monthly at either the VA or BU.
- Please Note: You will be updated with these events via email.

Photographs will be taken at the orientation of me. I agree to let these photos be used for educational purposes, publications, and presentations. I agree to be supervised by the study staff. The study staff will provide support and guidance and be available to me should a need arise. I agree to contact one of the program faculty or staff members immediately if issues of concern arise. After I

have successfully completed the Ambassador program, I will receive a letter of commendation from Neil Kowall, M.D, Director of the Boston University Alzheimer's Disease Center.

Only students who are able to honor their commitment to the program requirements should participate. We ask that interested students think carefully about whether they are able to fulfill the study commitments for the entire academic year. Our ability to engage with the community is impacted when a student drops out, so we ask that you take this commitment seriously.

Please sign below if you are able to make this commitment.

____

Student's Signature   Date

____

Student's Name (Please Print)

____

Program Coordinator's Signature   Date

____

Program Coordinator's Name (Please Print)
