# Supplementary material for: Educating students while recruiting underrepresented populations for Alzheimer’s disease research: the Student Ambassador Program
Source: BMC Med Educ. 2022 Oct 5;22:707. doi: 10.1186/s12909-022-03749-1 (PMC9533970; doi:10.1186/s12909-022-03749-1)
Supplement: Supplementary file 2 — Additional file 2. Boston University Alzheimer's Disease Center PAIRS Program Dementia Knowledge Test. [file 12909_2022_3749_MOESM2_ESM.pdf]

Boston University Alzheimer's Disease Center PAIRS Program
Dementia Knowledge Test

Please circle the most appropriate choice.

1. What is the greatest risk factor for Alzheimer's disease?
    a. **advancing age**
    b. having an immediate family member with Alzheimer's disease
    c. drinking from aluminum cans or cooking in aluminum pots
    d. apolipoprotein E (APOE) $\epsilon 4$ status
2. One in_persons age 65 and over has Alzheimer's disease.
    a. two
    b. **eight**
    c. twenty
    d. forty
3. Familial Alzheimer's disease (FAD) is generally clinically and histologically indistinguishable from the typical forms of Alzheimer's disease except
    a. there is more severe memory impairment in FAD
    b. **FAD typically begins at a younger age**
    c. there are fewer amyloid beta plaques in FAD patients
    d. FAD affects a greater number of cognitive domains, including language and executive function
4. Typical symptoms of Frontotemporal dementia include
    a. visual hallucinations and tremors
    b. difficulty with movement and coordination
    c. **changes in personality and behavior and difficulty with language**
    d. all of the above
5. The least common form of dementia is
    a. Lewy body disease
    b. Vascular dementia
    c. Alzheimer's disease
    d. **Frontal-lobe dementia**
6. The major histopathologic features of Alzheimer's disease are
    a. neurofibrillary tangles
    b. amyloid beta peptide deposition in senile plaques and blood vessels
    c. neuronal death
    d. **all of the above**
7. Deterministic mutations for familial Alzheimer's disease have been found on regions of the
    a. neuronal sortilin-related receptor (SORL1) gene
    b. apolipoprotein E (APOE) gene
    c. **presenilin-1 and presenilin-2 genes**
    d. all of the above

8. Potentially reversible causes of dementia include
    a. **Depression**
    b. Vascular dementia and mixed dementias
    c. Lewy body disease
    d. all of the above
9. Behavioral symptoms that may be present in moderate or advanced Alzheimer's disease are
    a. hallucinations and delusions
    b. eating and sleeping difficulties
    c. physical and verbal agitation
    d. **all of the above**
10. Adults with one parent with familial Alzheimer's disease have a chance of developing Alzheimer's themselves.
    a. 0%
    b. 25%
    c. **50**%
    d. 100%
11. _________ has sometimes been referred to as "chronic dementia" due to observations of reduced acetylcholine synthesis and devastation of cortically projecting cholinergic cells.
    a. **Alzheimer's disease**
    b. Frontotemporal dementia
    c. Vascular dementia
    d. Dementia with Lewy bodies
12. The term 'sundowning" refers to the
    a. excessive sleepiness experienced by some persons with moderate Alzheimer's disease
    b. **increased confusion, anxiety, agitation, and disorientation of some Alzheimer's patients that begins at dusk and continues into the night**
    c. nighttime hallucinations and wandering experienced by some late-stage Alzheimer's patients
    d. all of the above
13. Seven out of ten persons with Alzheimer's disease are cared for
    a. **at home**
    b. in nursing homes
    c. in assisted living facilities
    d. none of the above
14. The most widely accepted mechanism for the development of Alzheimer's disease is that the generation and deposition of amyloid beta leads to
    a. tangles
    b. oxidative stress
    c. inflammation and cell death
    d. **all of the above**

15. The largest group of informal caregivers for persons with Alzheimer's disease are
    a. **spouses**
    b. daughters
    c. sons
    d. sisters and brothers
16. Which type of dementia involves symptoms such as recurrent visual hallucinations and motor features of Parkinsonism?
    a. Alzheimer's disease
    b. Frontotemporal dementia
    c. Parkinson's disease
    d. **Dementia with Lewy bodies**
17. A definite diagnosis of Alzheimer's disease can only be reached through
    a. neuropsychological evaluation
    b. **autopsy**
    c. neuroimaging (CT, PET, MRI)
    d. clinical examination and history
18. Currently available pharmacologic treatments to slow the progression of Alzheimer's disease include
    a. acetylcholine antagonists
    b. norepinephrine and dopamine reuptake inhibitors
    c. **NMDA receptor antagonists**
    d. all of the above
19. A family history of dementia increases the relative risk of dementia by ______ times for persons up to age 80.
    a. 2
    b. **3-4**
    c. 10
    d. zero
20. The morphologic and biochemical origins of Alzheimer's disease are present in the early years of individuals with
    a. **Down syndrome**
    b. Moyamoya disease
    c. Cerebral palsy
    d. all of the above
21. Memory loss is a natural part of aging. (T / **F**)
22. Language difficulties, visuospatial problems, and executive functioning deficits are all symptoms of Alzheimer's disease. (**T** / F)
23. High blood pressure high cholesterol, and type 2 diabetes are all risk factors for cognitive decline. (**T** / F)
24. In order to be diagnosed as having dementia, the decline in cognitive abilities must be severe enough to interfere with daily life. (**T** / F)
25. Caucasians are more likely than African Americans to develop Alzheimer's disease. (T / **F**)

26. The presence of a single apolipoprotein E ϵ4 allele triples the risk of developing Alzheimer's disease. (**T** / F)
27. Approximately a half million Americans under the age of 65 have dementia. (**T** / F)
28. Having silver dental fillings could slightly increase your risk of developing Alzheimer's disease. (T / **F**)
29. Men are at a higher risk for Alzheimer's disease than women. (T / **F)**
30. Mental inactivity during midlife is associated with an increased risk for Alzheimer's disease. (**T** / F)
31. All cases of "mild cognitive impairment" typically eventually progress to Alzheimer's disease. (T / **F)**
32. There are treatments available to stop the progression of Alzheimer's disease. (T / **F**)
33. A history of depression may increase the risk of developing Alzheimer's disease later in life. (**T** / F)
34. Among persons over age 65, Alzheimer's disease is the 5th leading cause of death. (**T** / F)
35. Family and other unpaid caregivers of persons with Alzheimer's disease and dementia often experience higher levels of stress hormones and reduced immune function. (**T** / F)
36. Studies have shown that exercise may reduce the risk of Alzheimer's disease by preventing amyloid beta deposition. **(T** / F)
37. The type of Alzheimer's that is caused by deterministic genes, familial Alzheimer's disease, accounts for 20% of all cases. (T / **F**)
38. Normal pressure hydrocephalus can be a reversible cause of dementia. (**T** / F)
39. Some people live with Alzheimer's disease for up to 20 years. **(T** / F)
40. Drinking out of aluminum cans and cooking in aluminum pots and pans may slightly increase the risk of Alzheimer's disease. (T / **F**)
41. Within 5 years of their diagnosis, around 60% of people with "mild cognitive impairment" will develop Alzheimer's disease. (**T** / F)
42. Alzheimer's disease is less common in cultures that eat a lot of fish. (**T** / F)
43. There are treatments available that will help delay the progression of cognitive decline for some people with Alzheimer's disease. (**T** / F)
44. Studies have shown that head injury does not increase your risk for Alzheimer's disease. (T / **F**)
45. A person with Alzheimer's disease will live 4-6 years on average. (**T** / F)
46. A nutritious diet, exercise, social engagement, and mentally stimulating pursuits may help reduce the risk of cognitive decline and Alzheimer's disease. (**T** / F)
47. Wandering and pacing are common behaviors of a person with Alzheimer's disease. (**T** / F)
48. Low education is a risk factor for Alzheimer's disease. (**T** / F)
49. After being diagnosed with "mild cognitive impairment", some people will revert back to normal cognition. (**T** / F)

50. In order to avoid stressful communication, it is helpful to speak to an Alzheimer's patient using logical reasoning. (T / **F**)
51. In general eating and lifestyle habits that are bad for your heart are also bad for your brain. (**T** / F)
52. Studies show that moderate drinking (2-3/day) is associated with worse cognition. (T / **F**)
53. Half of all persons age 85 and older have Alzheimer's disease. **(T** / F)
54. Being fat in your 40s can raise your risk of developing dementia later in life. (**T** / F)
55. Almost all cases of Alzheimer's disease will first go through a prodromal stage of the disease, which is called "mild cognitive impairment". (**T** / F)
56. Redirecting and validation therapy are helpful techniques to use in communication with cognitively impaired persons. (**T** / F)
57. Universal evidence has shown that elevated lipids and cholesterol are risk factors for Alzheimer's disease. (T / **F**)
58. Depressive symptoms often coexist with dementia. (**T** / F)
59. Early-stage and moderate Alzheimer's disease patients often have the most difficulty remembering long-term memories, as opposed to new information. (T / **F**)
60. One third of people who have Parkinson's disease will develop dementia in the later stages of the disease. (**T** / F)
61. Social and conversational skills are often so well preserved in someone with mild or early-stage Alzheimer's disease that these patients often appear normal to the casual observer. (**T** / F)
62. Stroke is associated with increased risk of Alzheimer's disease and cognitive decline. (**T** / F)
63. An electroencephalogram (EEG) is a useful routine diagnostic tool for the evaluation of cognitive impairment in older persons. (T / **F**)
64. Overmedication is a reversible cause of dementia. (**T** / F)
